# Sphenoid Meningoencephalocele Correction Through a Transpterygoid Approach

**DOI:** 10.7759/cureus.52555

**Published:** 2024-01-19

**Authors:** Fernando M Mar, José Miranda, António F Lima, Guilherme Rios, Luís Dias

**Affiliations:** 1 Otorhinolaryngology and Head and Neck Surgery Department, Hospital de Braga, Braga, PRT

**Keywords:** transpterygoid endonasal approach, skull base defect, endoscopic endonasal surgery, cerebrospinal fluid leak, sphenoid meningoencephalocele

## Abstract

Sphenoid meningoencephaloceles are rare, and their treatment is challenging. In this report, we describe two clinical cases of sphenoid meningoencephalocele, in which one patient presented with a cerebrospinal fluid leak after repeated head trauma, while in the other, sphenoid meningoencephalocele was detected during the study of memory impairment as the patient was otherwise asymptomatic. The CT scans showed bony dehiscence on the lateral wall of the sphenoid sinus filled with soft tissue that was confirmed by MRI as being herniated brain tissue. A transpterygoid endoscopic endonasal approach was performed with a multilayer reconstruction of the defect with success in both cases without perioperative complications. Imaging techniques are fundamental for diagnosis and surgical planning. Treatment using an endoscopic endonasal approach is efficient with very low morbidity.

## Introduction

Skull base defects may originate a communication between the intracranial cavity and a pneumatized structure of the skull base such as the sinonasal, the middle ear, or the mastoid space, possibly leading to a cerebrospinal fluid fistula or a herniation of intracranial tissue, namely a meningocele or a meningoencephalocele [1]. If left untreated serious complications such as central nervous system infection may arise. Treatment is challenging and depends on the bony defect location and its dimensions [2]. Sphenoidal meningoencephaloceles are rare; in this case report we summarize two clinical cases with completely different etiology and clinical presentation. We show the efficacy of endoscopic endonasal correction and its low morbidity.

## Case presentation

Clinical case 1

The first case refers to a 65-year-old female with a one-year history of right-sided, clear, watery rhinorrhea that increased during a Valsalva maneuver. Besides a history of breast cancer treated with surgery and chemotherapy 11 years before, well-controlled hypertension, and being overweight, the patient reported previous repeated minor accidental head trauma.

The clinical examination confirmed the unilateral clear watery rhinorrhea; no additional findings were observed on the nasal endoscopy. No specific analysis of the nasal fluid was performed.

**Figure 1:**
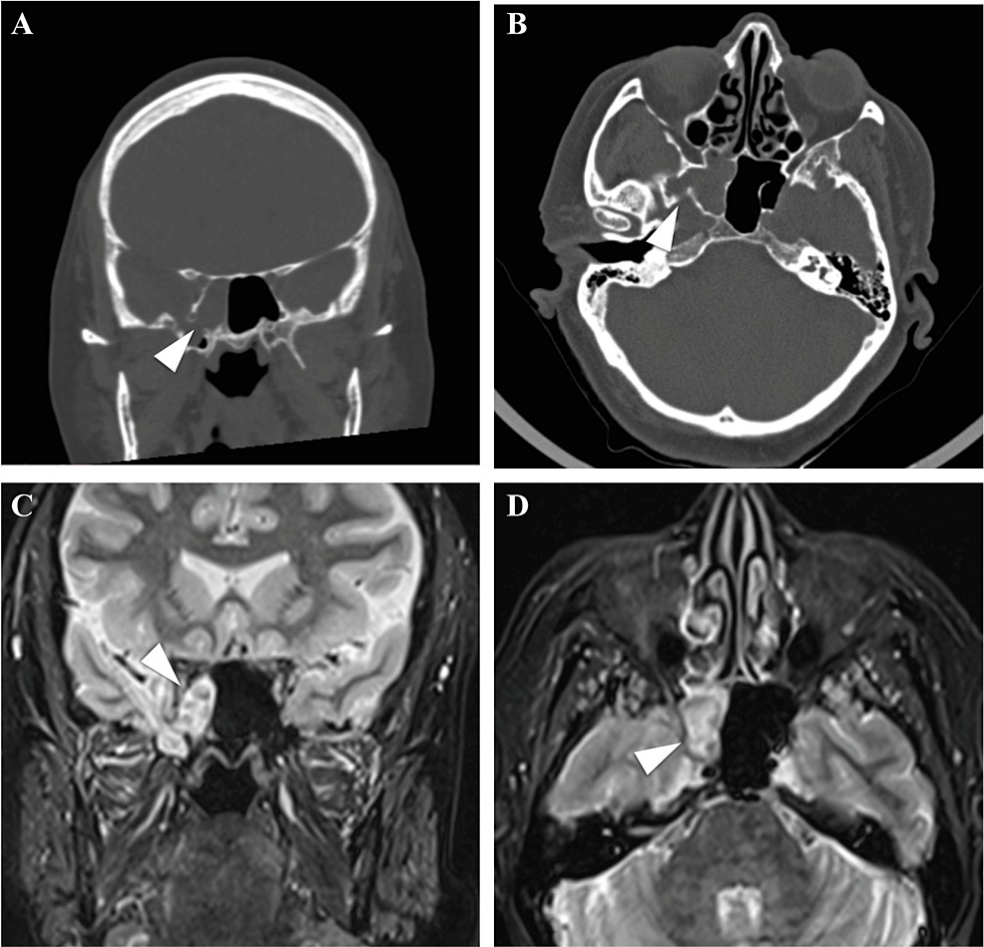
CT scans and MRI confirm inferolateral sphenoid wall dehiscence and brain herniation. CT scans (A and B) show a dehiscence of the inferolateral sphenoid wall, establishing communication with the middle cranial fossa (arrowheads). MRI (C and D) shows bone discontinuity in the right lateral wall of the sphenoid sinus and in the ipsilateral petrous apex, crossed by tissue with signal emission characteristics similar to brain parenchyma and CSF, involving the right chamber of the sphenoid sinus, the ipsilateral Meckel cave, and the base of the pterygoid processes bilaterally (arrowheads).

The patient underwent both CT scans and an MRI. The CT scans showed an opacification of the right sphenoid and a possible dehiscence of the inferolateral sphenoid wall, possibly the Stenberg’s canal (Figure 1A, 1B). The MRI confirmed the presence of meningoencephalic herniation through the right lateral sphenoid wall and petrous bone involving also the Meckel’s cave (Figure 1C, 1D).

**Figure 2:**
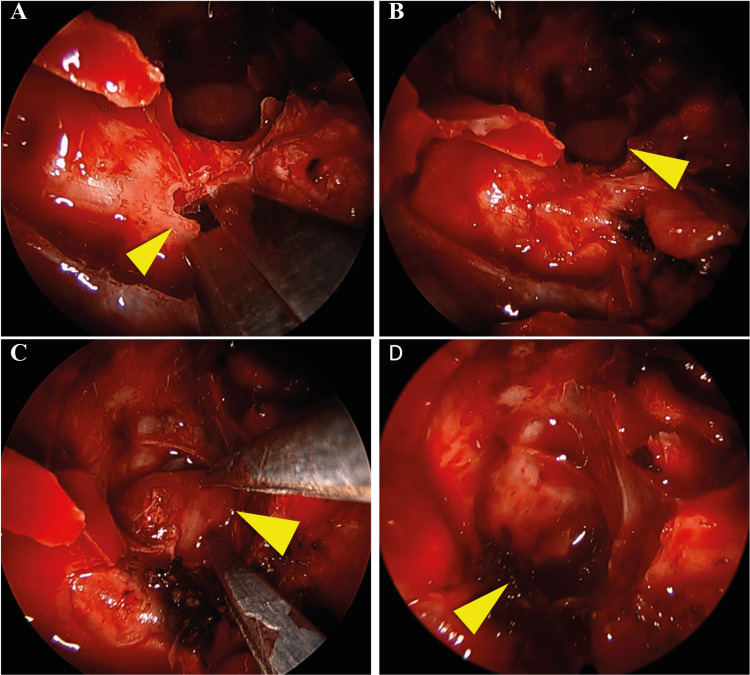
Transpterygoid approach to access sphenoid meningoencephalocele. The posterior wall of the maxillary sinus was exposed and removed (arrowhead, A). The herniated brain was then exposed (arrowhead, B). The meningoencephalocele was then retracted with bipolar cauterization (arrowhead, C). Following the retraction, no herniated brain tissue was observed in the sphenoid sinus (arrowhead, D).

The defect was surgically ablated, through an endoscopic endonasal approach. The skull base defect was accessed through a transpterygoid approach. The maxillary sinus was exposed by removing its medial wall, the sphenopalatine foramen was identified, and the sphenopalatine artery was cauterized and sectioned. A complete ethmoidectomy and sphenoidectomy were performed. At this point, the brain herniation was identified on the right lateral wall of the sphenoid sinus. The posterior wall of the maxillary sinus was then removed until the infra-orbitary nerve. The pterygoid process was then identified and partially removed and the dehiscence with herniated tissue was identified. The herniated brain tissue was bipolar retracted, and a multilayer reconstruction was performed with fat tissue, fascia lata, and nasoseptal flap (Figure 2).

**Figure 3:**
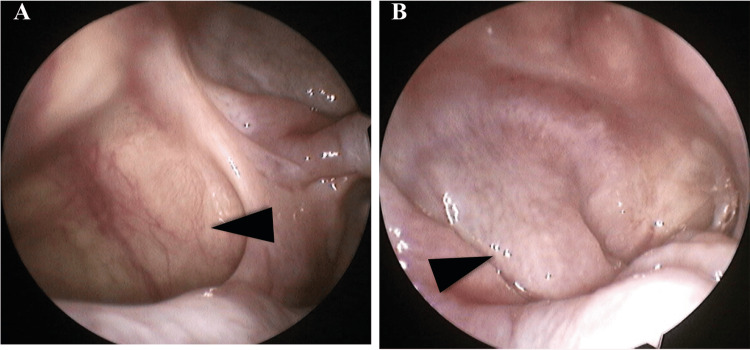
Post-surgical evaluation shows a completely healed nasal cavity. The maxillary sinus (arrowhead, A) and the sphenoid sinus (arrowhead, B).

The perioperative period was uneventful, allowing a complete nasal cavity healing (Figure 3).

After four years of follow-up, the patient remains symptom-free.

Clinical case 2

**Figure 4:**
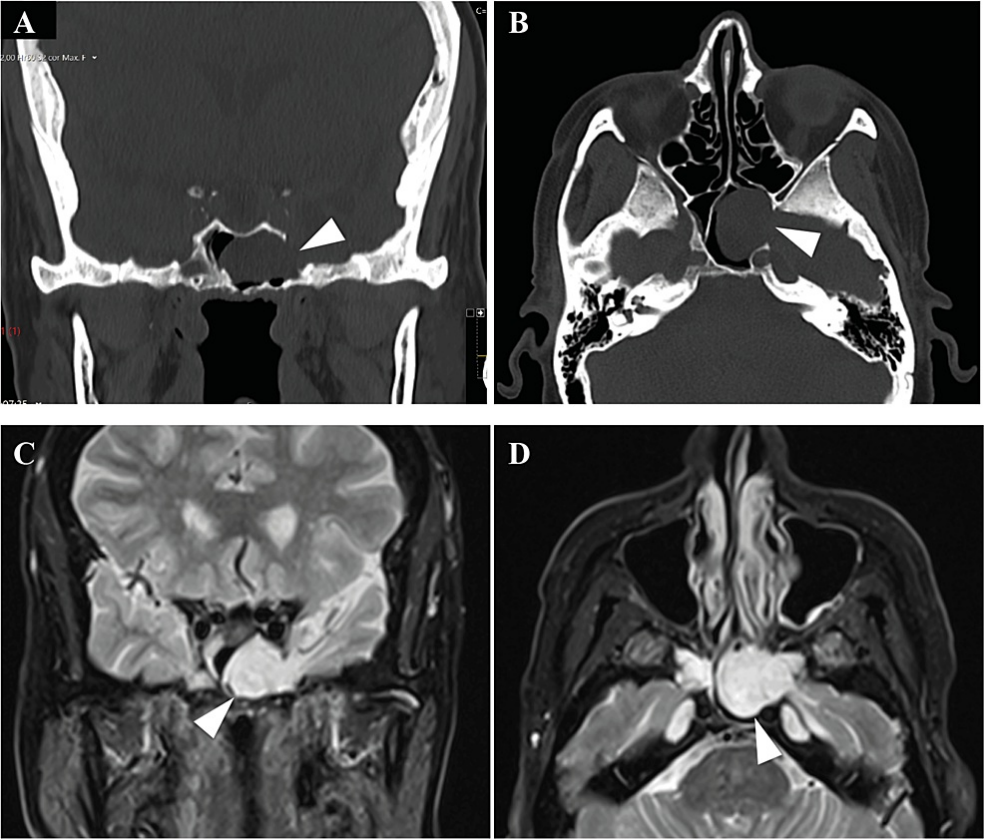
CT and MRI show dehiscence of the lateral left sphenoid wall with brain herniation. CT scans (A and B) show dehiscence of the lateral left sphenoid wall (arrowheads), establishing communication with the middle cranial fossa. MRI (C and D) shows bone discontinuity in the left lateral wall of the sphenoid, crossed by tissue with signal emission characteristics similar to brain parenchyma and CSF to the left chamber of the sphenoid sinus (arrowheads).

A 73-year-old female with diabetes, hypertension, and dyslipidemia medically controlled underwent a CT scan in the study of mild memory impairment. The CT scan showed an opacification of the left sphenoid sinus and the absence of the left lateral sphenoid wall (Figure 4A, 4B). The patient was then referred to ENT observation. She did not have any otorhinolaryngological complaints; the physical examination, including the nasal endoscopy, was normal. The imaging study was complemented with an MRI that showed an encephalocele through the left lateral sphenoid wall to the sphenoid sinus (Figure 4C, 4D).

**Figure 5:**
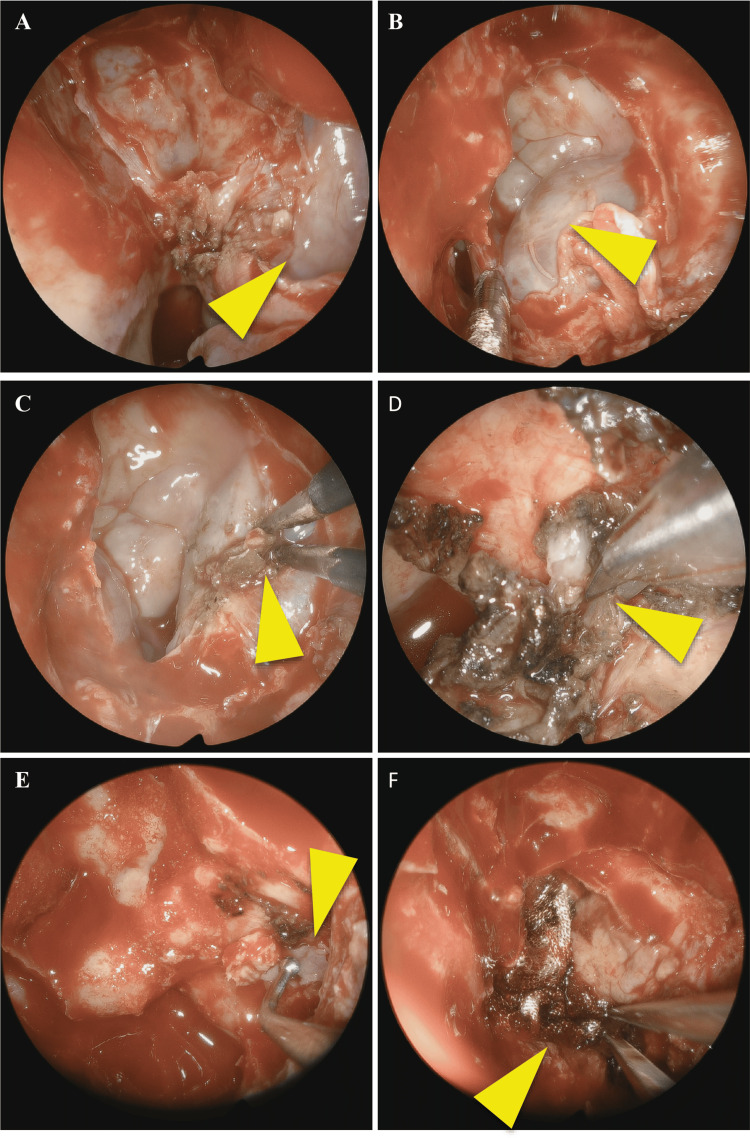
A transpterygoid endonasal approach with multilayer reconstruction was used. The pterygoid fossa was exposed maintaining its periosteum (arrowhead, A). Following sphenoidotomy, the meningoencephalocele was exposed (arrowhead, B), and then shrunk with bipolar cauterization (arrowhead, C). The pedicle of the herniated tissue was then sectioned (arrowhead, D). The defect of the lateral wall of the sphenoid sinus was then reconstructed with bone from the middle turbinate (arrowhead) and oxidized regenerated cellulose (E). Finally, a nasoseptal flap was used to cover the sphenoid with another layer of oxidized regenerated cellulose (arrowhead, F).

An endoscopic endonasal approach was used to ablate the defect. A maxillary antrostomy followed by a complete ethmoidectomy and sphenoidectomy was performed. The pterygopalatine fossa was exposed, maintaining the periosteum intact. The meningocele was later identified on the left sphenoid sinus. The meningocele was retracted through bipolar cauterization until the level of the bone dehiscence and then resected. A multilayer reconstruction was done using oxidized regenerated cellulose, a bone from the middle turbinate, a dura substitute, and a nasoseptal flap (Figure 5A-5F).

No perioperative complications were observed, and after one year of follow-up, the patient remains asymptomatic.

## Discussion

Sphenoid meningoencephalic herniation is more often an incidental imaging study finding. Classically it has been proposed as a consequence of a persistent Sternberg canal resulting from an incomplete fusion of the greater wings of the sphenoid bone with the basisphenoid [3,4]. However, the presence of a Sternberg canal is not as frequent as initially reported [5]. Other physiologic and anatomical alterations like an empty sella, or arachnoid pits have been proposed to contribute to this pathology [6-8].

CSF fistulas can be classified according to their cause. They are usually divided as acquired or congenital. In the acquired group, they can be further divided into traumatic (accident or iatrogenic trauma as a result of surgery), non-traumatic (tumors or inflammation/infection), or idiopathic/spontaneous. Spontaneous fistulas are usually associated with obesity and increased intracranial hypertension [1]. The increased intracranial hypertension may contribute to the formation of the arachnoid pits that can result in focal erosions of the skull base, culminating in dural protrusion and brain herniation [5,8]. Trauma is the most common cause of CSF fistulas. In this report, patient 1 reported previous repetitive head traumas, making it the most probable cause of the meningoencephalocele, while in patient 2, no probable cause was identified, which led us to classify the meningocele as idiopathic.

Settecase et al. proposed a classification for the lateral sphenoid meningoencephaloceles, resulting in two types. In type 1 there is a herniation into the lateral recess of the sphenoid sinus, and in type 2 there is a herniation into the greater wing of the sphenoid. Usually, this results in different clinical presentations. While the clinical presentation in type 1 patients is usually a CSF leak and/or headache, type 2 patients commonly have seizures and/or headaches, but they can also be asymptomatic [9]. In both presented clinical cases, the patients have a type 1 alteration. While in patient 1 the CSF fistula led to the diagnosis, without any other complaints, patient 2 was completely asymptomatic.

The imaging studies are central in the evaluation of the patient with a suspected skull base defect. High-resolution CT scan together with MRI may be sufficient to identify the defect and the herniated tissue. However, other methods may be used. CT and/or MRI cisternography may be necessary if the previous exam fails to identify the precise location of the skull defect [9-11]. Non-imaging techniques may contemplate the identification of β2-transferrin on rhinorrhea, as it confirms its CSF origin [3,9]. In the case presented here, the diagnosis was made with a high-resolution CT scan and MRI. The β2-transferrin identification assay is not available in our hospital. The intrathecal administration of fluorescein intra-operatively has been used to identify the leakage site; however, the dosage used may vary considerably among institutions [12]. Also, it has not been used in any of the cases described in this report.

The correction of the encephalocele/CSF should be performed early, so infectious complications such as meningitis, encephalitis, or brain abscess can be prevented [9]. The goal of the surgery is the resection of the encephalocele, as herniated tissue is considered functionless, and the closure of the defect. With the advance of endoscopic surgery, transcranial approaches were replaced by endoscopic endonasal surgery due to its lower morbidity. The exception may be CSF laterally or from the posterior wall of the frontal sinus [13,14]. The endoscopic transpterygoid approach has the advantage of avoiding craniotomy and brain retraction; however, it may result in neurovascular injury of the pterygopalatine fossa content during the access to the lateral recess of the sphenoid sinus [3,11]. The skull base reconstruction can be performed using different techniques and materials; it is still controversial which presents the best outcomes [11,15]. The location and size of the defect must be taken into account for the decision.

While it is widely accepted that the endoscopic correction of sphenoid meningoceles has lower morbidity, the data on the success of this approach as well as its comparison with a transcranial approach is scarce. Two of the largest case series on endoscopic correction report success rates of 92-100% [16,17]. Here, we describe two cases that were successfully corrected.

## Conclusions

Sphenoid encephaloceles are rare and often incidental imaging study findings. As described in this report, a multilayer reconstruction through an endoscopic transpterygoid approach can be safely used to treat sphenoid meningoencephalocele. Perioperative complications and recurrence rates are rare in the endoscopic endonasal approach.
